# Effect of Brisk Walking on Health-Related Physical Fitness Balance and Life Satisfaction Among the Elderly: A Systematic Review

**DOI:** 10.3389/fpubh.2021.829367

**Published:** 2022-01-31

**Authors:** Xiaorong Bai, Kim Geok Soh, Roxana Dev Omar Dev, Othman Talib, Wensheng Xiao, Haogang Cai

**Affiliations:** ^1^Department of Sports Studies, Faculty of Educational Studies, Universiti Putra Malaysia, Seri Kembangan, Malaysia; ^2^Department of Science and Technical Education, Faculty of Educational Studies, Universiti Putra Malaysia, Seri Kembangan, Malaysia; ^3^School of Physical Education, Shangqiu Normal University, Shangqiu, China

**Keywords:** cardiorespiratory fitness, muscular endurance, muscular strength, body composition, flexibility, balance, life satisfaction

## Abstract

**Background:**

Although the elderly frequently engages in brisk walking as a form of exercise, little has been reported in the literature about the effect of brisk walking on health-related physical fitness, balance, and overall life satisfaction.

**Objectives:**

The purpose of this systematic review is to determine the effect of brisk walking on the elderly's health-related physical fitness, balance, and life satisfaction.

**Design:**

We conducted a comprehensive search from the PubMed, Web of Science, Scopus, and SPORTDiscus databases from January to September 2021. We selected studies through PICOS and conducted a systematic literature review according to the PRISMA guidelines.

**Results:**

Thirteen studies met all criteria; 11 were classed as low risk of bias, while two were classified as high risk of bias. Generally, brisk walking has been shown to improve cardiorespiratory fitness, muscular strength, and body composition. Limited evidence was presented on flexibility, muscular endurance and development and life satisfaction, and there was conflicting evidence on balance. Moreover, evidence of restriction proves that high-intensity (80–85%) brisk walking is more effective than moderate-intensity (60–75%) brisk walking on the aerobic capacity of the elderly. Furthermore, there was less research conducted on males.

**Conclusion:**

Brisk walking has been shown to improve cardiorespiratory fitness, muscular strength, and body composition. Other outcomes (balance, flexibility, muscular endurance, and life satisfaction) and the impact of the intensity of brisk walking on the elderly should be confirmed. Therefore, there remains insufficient research on brisk walking, while single brisk walking cannot meet requirements of elderly in terms of their health-related physical fitness, balance, and life satisfaction. Future research should aim to examine the effectiveness of combining several types of exercises to promote general health in the elderly, as the World Health Organization recommends. Unintelligible FITT (frequency, intensity, time, type) principles of brisk walking training should be trenched for the results of scientific and effective physical exercise.

## Introduction

As a result of the global aging trend, many countries have increased their focus on the health of the elder population (1). Physical exercise has been shown to improve the overall health and quality of life (QoL) of the elderly by enhancing their physical and mental health (2–4). Walking is a low-cost, low-impact form of exercise. Not only does it improve general health and improve QoL, but it also plays a critical role in the prevention and treatment of numerous diseases (5).

Age is a significant risk factor for non-communicable chronic diseases (NCDs) such as chronic obstructive pulmonary disease, cardiovascular disease (CVD), type 2 diabetes, cognitive decline, dementia, and cancer (6), all of which have high associated diagnostic, treatment, and care costs. According to the WHO, more than 80% of the elderly suffer from at least one NCDs (7). Cardiovascular diseases account for most NCD deaths, or 17.9 million people annually (8). Moreover, falls are the second greatest cause of unintentional injury deaths. The risk of death or serious injury is greatest for older adults. Furthermore, some studies have shown that the worse the physical health, the lower the mental health score (9, 10). Mental health and physical health are equally for elderly, which influence greatly an overall feeling of well-being (8). Thus, the main issues of elderly include cardiovascular diseases, falls, and mental health. The World Health Organization (WHO) recommends ~150 min of moderate-intensity physical activity or exercises per week to reduce the risk of death from any cause, cardiovascular disease, hypertension, site-specific cancers, type-2 diabetes, prevention of accidental falls, decreased mental health (anxiety and depression symptoms), cognitive health, and measures of adiposity (7). Thus, supporting healthy aging through physical activity, self-sufficiency, and leisure time becomes a critical public health problem for enhancing an individual's health (11). Walking is among the most popular physical exercises on a global scale (12). It features simple and natural movements, a moderate level of workout intensity, and a long lifespan. Meanwhile, it has the advantage of individuals being less prone to injury and posing little risk, making it an excellent choice for middle-aged and elderly individuals (13). Moreover, previous study have presented that walking can reduce falls times (14), prevent cardiovascular disease (15), ease up anxiety, and depression (16). Thus, walking may become one of the effective exercise methods to alleviate the current health-related problems faced by the elderly. Base on a book of ACSM's exercise for older adults, FITT (frequency, intensity, time, type) principle should be followed when elderly exercise, which improve physical fitness effectively (17). Generally, elderly people who participate in exercise are not sure how fast walking will have a greater impact on the health. Therefore, it is necessary to review the literature to sort out what exercise principles are most effective in promoting the health of the elderly.

Through combing the effects of brisk walking on the health of the elderly, discovered that most researchers only study its effects on part of the physical fitness such as lower body strength (18, 19), cardiorespiratory fitness (20–23), body composition (24), no studies mantled all components, and penurious researches on life satisfaction. Not only that some studies have unclear reports on exercise intensity (19, 25, 26). Moreover, only two papers contrast in brisk walking intensity (24, 27). Additionally, identifying workouts that have a greater influence on health-related physical fitness, balance, and life satisfaction to prevent and defend against the emergence of health problems warrants further research. The President Council on Physical Fitness has defined health-related physical fitness as “those specific components of physical fitness that have a relationship with good health” (28). Cardiorespiratory fitness, body composition, flexibility, muscular strength, and muscular endurance are all components of health-related fitness. Physical fitness has been demonstrated to be a strong independent predictor of death in previous research (29). Among them, improving cardiovascular fitness can reduce your risk of developing cardiovascular disease by increasing the efficiency of your heart, lungs, and blood vessels (30). Additionally, balance exercises are critical in avoiding elderly people from falling (31). Life satisfaction is more important to the mental health of the elderly (32). Hence, highly related to health-related physical fitness, balance and life satisfaction, and health-related issues of the elderly. According to the health-related fitness model (33), physical activity can promote health-related fitness eventually affect health which includes wellness, mortality, and morbidity. In this study, brisk walking as a physical activity to improve elderly health-related fitness and balance eventually affect health. Additionally, Life satisfaction is regarded as an indicator of the quality of life which, in turn, is associated with mortality (34–36) and morbidity in older adults (37, 38). Given that brisk walking is a necessary type of exercise for the elderly, what are the impacts on their physical health? Is it effective at preventing and protecting against a variety of chronic diseases that affect the elderly? As such, the purpose of this study is to determine the effect of brisk walking on the health-related physical fitness, balance, and life satisfaction of senior individuals.

## Materials and Methods

### Eligibility Criteria

A systematic review was carried out according to the Preferred Reporting Items for Systematic Reviews and Meta-analyses (PRISMA) statement. Table 1 summarizes the inclusion criteria for this review, which are Population, Intervention, Comparison, Outcome, and Study Design (PICOS). In addition to the above screening criteria, studies were included if they satisfied the following criteria: (1) full-length, peer-reviewed journal articles; (2) healthy study participants (excluding those who were obese or weak); and (3) consideration of objective measurements of health-related physical fitness, balance, and life satisfaction. Physical fitness for health is defined as a subset of fitness that encompasses cardiorespiratory fitness, muscular strength, muscular endurance, flexibility, and body composition. These components are related with daily activities and are critical for older individuals to preserve their independence (39). Balance and life satisfaction are related with falls and the mental health of the elderly. Additionally, they entail moderate and vigorous walking, but not running or Nordic walking. To eliminate duplication, the considered studies were loaded into Mendeley's reference management program. To begin, an experimental librarian led the search procedure. Second, the titles and abstracts were independently reviewed by two reviewers. Following that, pertinent full-text articles were selected for further investigation. In the event of any disagreement between two reviewers, a third reviewer served as a tiebreaker.

**Table 1:** Population, intervention, comparator, outcome, and study design (PICOS) detail screening criteria.

**PICOS**	**Screening criteria**
Participants	Healthy adults, age ≥ 60 yrs old (except obesity, frail, cancer, and other diseases)
Intervention	It must be a moderate and vigorous walking, excluding running and Nordic walking.
Comparison	No exercise group or exercise group in the control group
Outcomes	Strength, endurance, balance, flexibility, body composition, cardiorespiratory fitness, balance, life satisfaction
Study design	Randomized controlled trial

### Data Sources and Search

A systematic search was undertaken on the existing literature on the impact of brisk walking on health-related physical fitness, balance, and life satisfaction in the elderly, published before December 2021. The study was designed and conducted in accordance with the PRISMA statement (40).

The literature search was conducted using four prominent scholarly databases: PubMed, Web of Science, Scopus, and SPORTDiscus. All keywords were searched by Mesh of PubMed and previous studies. Each database was searched by title using a predefined combination of keywords (“health-related physical fitness” OR “physical fitness” OR “muscular strength” OR “muscular endurance” OR “flexibility” OR “body composition” OR “cardiorespiratory” OR “balance” OR “satisfaction with life” OR “life satisfaction”) AND (“brisk walk^*^” OR “moderate-intensity walk^*^” OR “high-intensity walk^*^”) AND (“old people” OR “elders” OR “senior^*^” OR “old adult^*^” OR “aged” OR “older people” OR “older adults” OR “geriatric”). Terms were joined with the use of logical operators that can be utilized by the database search engines. Additionally, the authors consulted experts in the field.

### Study Selection

An author conducted a search for articles and deleted duplicates. Two authors independently chose studies based on their titles and abstracts. If this was unsuccessful, the papers were screened by reading the complete text. The following information was extracted: (1) author/year; (2) design/sample/age/gender; (3) intervention time/frequency/duration; and (4) major findings.

### Quality Assessment

The PEDro scale was used to assess the trials' methodological quality (41). The PEDro scale assesses four critical methodological features of a study: randomization, blinding, group comparison, and data analysis. It is based on a Delphi list developed by Verhagen et al. (42), which includes the following 11 items: specified eligibility criteria, randomization, concealed allocation, baseline comparability, blinded subjects, blinded therapists, blinded assessors, adequate follow-up, intention-to-treat analysis, between-group comparisons, and point estimates and variability. Two trained independent raters assessed the quality of trials in the PEDro database, and conflicts were settled by a third rater (43). The PEDro scale has a score range of 1 to 10; whereby a higher PEDro score indicates a higher-quality approach. To determine the method's quality, the following criteria were used: A PEDro score of <5 denotes poor quality, while a score of 5 or greater indicates excellent quality (Table 2) (47).

**Table 2:** Summary of methodological quality assessment scores.

**References**	**Eligibility criteria**	**Random allocation**	**Allocation concealment**	**Group similar at baseline**	**Blind subject**	**Blind therapist**	**Blind assessor**	**Follow-up**	**Intention to Treat Analysis**	**Between group comparisons**	**Point measure and variability**	**PEDro score**
Audette et al. (18)	1	1	0	1	0	0	0	1	0	1	1	6
Bernard et al. (20)	1	1	0	1	0	0	0	1	0	1	1	6
Blain et al. (21)	1	0	0	1	0	0	0	1	0	0	1	4
Fisher and Li (25)	1	1	0	1	0	0	0	1	0	1	0	5
Kubo et al. (19)	1	1	0	1	0	0	0	1	o	1	1	6
Morita et al. (22)	1	1	0	1	0	0	0	1	0	1	0	5
Nemoto et al. (27)	1	1	1	1	0	0	0	1	0	1	1	6
Okubo et al. (23)	1	1	0	1	0	0	0	1	0	1	0	5
Paillard et al. (26)	1	1	1	1	0	0	0	1	0	0	1	6
Song et al. (44)	1	1	0	1	0	0	0	1	0	1	0	5
Swoap et al. (24)	1	0	0	0	0	0	0	1	0	0	1	3
Wanderley et al. (45)	1	0	1	1	0	0	0	1	0	0	1	5
Yoo et al. (46)	1	1	1	0	0	0	0	1	0	0	1	5
**Total**	**13**	**10**	**4**	**11**	**0**	**0**	**0**	**13**	**0**	**8**	**9**	

*The bold values means summary of the quality evaluation of all papers*.

### Data Syntheses and Analysis

This study is a Meta-aggregation of Qualitative Data Synthesis. The scientific evidence's strength was determined by utilizing the most effective evidence synthesis (BES). This evaluation system considers the quantity, methodological quality, and consistency of research across five levels of evidence: (1) strong evidence, provided by generally consistent findings in multiple (≥2) high-quality studies; (2) moderate evidence, provided by generally consistent findings in one high-quality study and one or more low-quality studies, or in multiple low-quality studies; (3) limited evidence, provided by only one study or inconsistent findings in multiple (≥2) studies; and (4) conflicting evidence, provided by conflicting findings in case–control studies (75% of the time) and (5) absence of evidence, in the absence of case–control research.

## Results

### Study Selection

**Figure 1:**
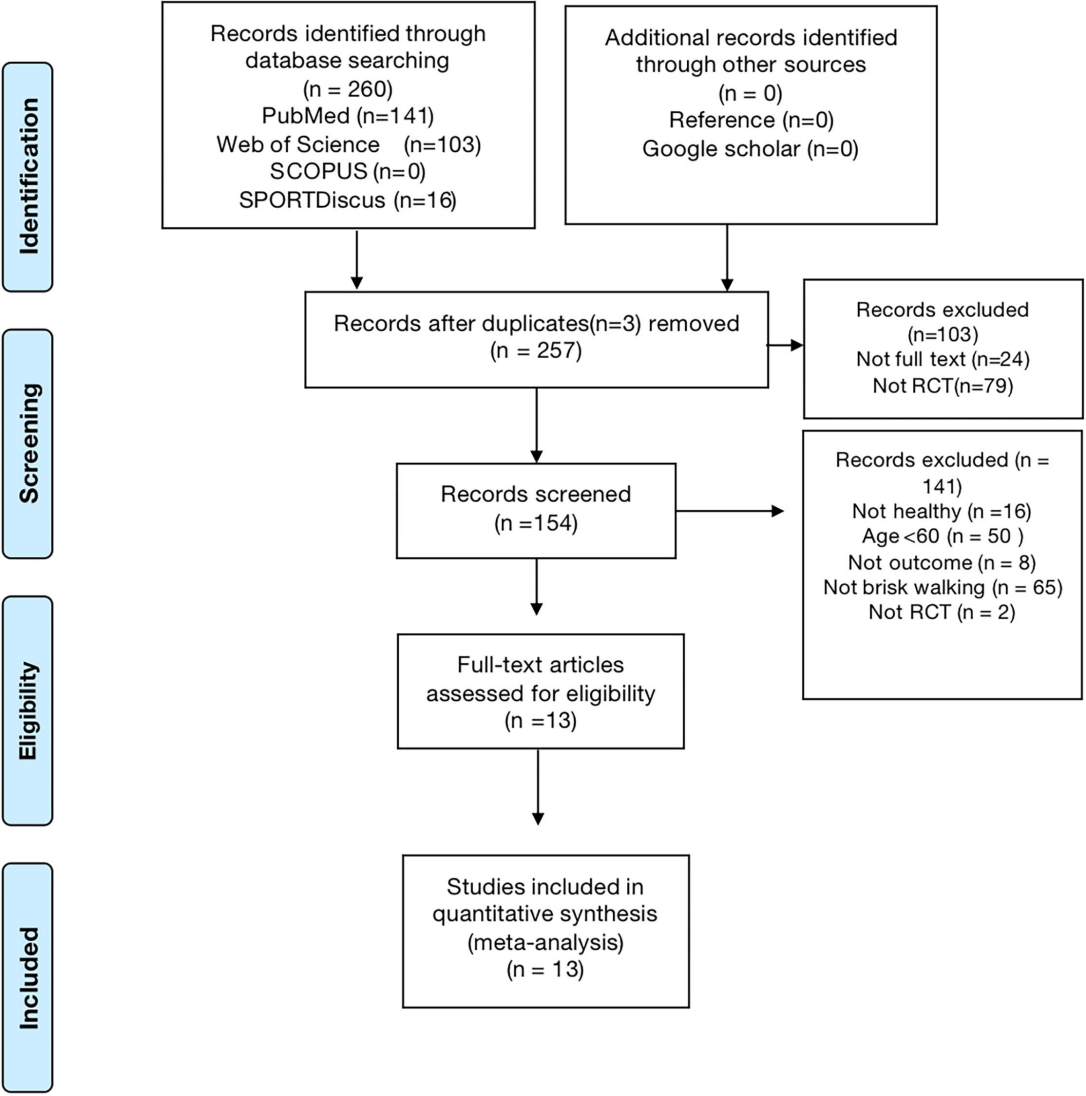
PRISMA flow chart of the study selection process.

The database search returned 260 records: 141 from PubMed, 103 from Web of Science, 0 from Scopus, and 16 from SPORTDiscus. Duplicate references were removed, resulting in a total of 257 articles. One hundred and fifty-four articles were eliminated from consideration for inclusion based on the topic and abstract screening. Finally, 13 articles that were deemed to be extremely relevant were analyzed by reading the complete text. Consequently, 13 articles were selected for inclusion in this systematic review. The process carried out for the study is depicted in Figure 1.

### Method Quality

The PEDro scale had a range of values between 4 and 7 (mean = 5.2; median = 5; mode = 5). Two studies received a score of <5, while the remaining 11 (*n* = 11) received a score of five or higher, indicating a mix of high- and low-quality studies. The publication year did not influence the quality of the studies, since the low-quality studies were published in 2016 and 2017, while the high-quality studies were published between 2004 and 2019 (see Table 2). The mostly met criteria were eligibility criteria (*n* = 13), group similar at baseline (*n* = 11), point measure and variability (*n* = 9), random allocation (*n* = 10), between-group comparisons (*n* = 8), and follow-up (*n* = 13). The criteria blind subject, blind therapist, blind assessor, and intention to treat analysis did not satisfy any analysis. In terms of concealed allocation, *n* = 4 (Table 2).

### Study Characteristics

The study characteristics included in this review are shown in Table 3. All studies were published between 2004 and 2019. In terms of the study design of pre- and post-test (*n* = 11) (18–20, 22–24, 26, 27, 45, 46, 48), one study tested four times (44) and one study tested three times (25). Regarding the participants, the studies included males (26) and females (18, 20–22, 44–46), or both sexes (19, 23–25, 27), with the mean value of ages ranging from 61.83 to 71.9. The sample size ranged from 9 to 68 participants. Brisk walking comprised an experimental group or a control group. The total intervention time ranged from 12 to 52 weeks, while the frequency ranged from 3 to 7, and the duration ranged from 15 to 60 min. There are five ways to assess intensity which ranged from 40% to 85% of heart rate reverse, 40%-80% of maximum heart rate, ≥ 3 metabolic equivalents (METs) daily (22), ~50% VO_2_ peak (27), and “Light (11)” and “Somewhat hard (13)” according to the perceived exhaustion scale (23).

**Table 3:** Characteristics of the studies examined in the present review.

**References**	**Design**	**Participants**	**Age mean ±SD (yrs)**	**Main section content (CG/EG)**	**Intervention**		**Findings**
					**Intensity**	**(Wk/f/min)**	
Audette et al. (18)	Pre-post-test	EG1 = 11 (F) EG2 = 9 (F) CG = 9 (F)	EG = 71.4 ± 4.5	EG1 = Tai Chi Chuan EG2 = Brisk walking CG = Sedentary comparison group	50–70% of their calculated target heart rate (220—age)	EG1 = 12/3/60 EG2 = 12/3/60	Significant improvement: non-dominant knee extensor strength ↑, single-leg stance time ↑ (Tai Chi Chuan vs. brisk walking, *P* < 0.05). No significant differences: on VO_2_ max ↔.
Bernard et al. (20)	Pre-test, post-test1, post-test 2	EG = 61 (F) CG = 60 (F)	EG1 = 65.54 ± 4.04 CG = 65.46 ± 4.37	EG = Brisk walking group CG = Control group	60–80% of their maximal heart rate calculated by equation of Tanaka [FC_max_ = 208 – (0.7 × age)]	EG1 = 24/3/60 (brisk walking from 25 to 45 min) CG = 24/3/60	Significant improvement: 6MWT between pre-test and post-test 1 ↑, between post-test 1 and post-test 2 ↑.
Blain et al. (21)	Pre-posttest	EG1 = 61 (F) CG = 60 (F)	EG = 65.7 ± 4.3	EG = Brisk walking CG = Control group with physical activity allowed freely	40 to 60–80% of maximal heart rate and was calculated using Tanaka's equation [208 – (0.7 × age)].	EG = 24/3/50 CG = 24/3/50	Significant improvement: walking endurance (6MWD) ↑.
Fisher and Li (25)	Pre-test, post-test 1, post-test 2	EG = 28 (M\F) CG = 28 (M\F)	EG>65 CG>65	EG = leader led walking group CG = Information group	No presented	EG = 24/3/30-40 CG = 24/3/30-40	Significant improvements: in the primary outcomes of SWLS (*p* < 0.05) ↑ between primary and 6-month intervention.
Kubo et al. (19)	Pre-posttest	EG = 35 (M\F) CG = 10 (M\F)	EG = 68.4 ± 5.6 CG = 71.9 ± 2.7	EG = Walking training CG = No exercise	No presented	EG = 24/3/15-40	Significant changes: lower limbs strength (KF, DF, PF) ↑.
Morita et al. (22)	Pre-posttest	EG1 = 18 (F) EG2 = 14 (F)	EG>65	EG1 = Aerobic exercise (brisk walking) EG2 = Muscles training	≥ 3 metabolic equivalents (METs) daily	EG1 = 12/7/60 EG2 = 12/7/60	Significant improvement: cardiorespiratory fitness (6MWT) ↑.
Nemoto et al. (27)	Pre-posttest	EG1 = (M = 16, F = 59) EG2 = (M = 19, F = 68) CG = (M = 25, F = 59)	EG = 63 ± 6	EG1 = Moderate-intensity continuous walking training EG2 = High-intensity interval walking training CG = No walking training	EG1 = Walking more than 8,000 steps per day at ~50% VO_2peak._ EG2 = low-intensity walking intervals (at ~40% of the pre-training VO_2peak_), followed by a 3-min interval of high-intensity walking (>70% but <85% VO_2peak_ for walking).	EG1 = 20/4/60 EG2 = 20/4/50	Significant changes: leg strength ↑, peak aerobic capacity ↑, and peak aerobic capacity ↑ were observed in the high-intensity interval walking training group. All these increases were significantly larger than those reported in the group receiving moderate-intensity continuous walking training.
Okubo et al. (23)	Pre-posttest	EG1 = 42 (F/M) EG2 = 33 (F/M)	EG1 = 70.3 ± 3.9 EG2 = 70.0 ± 3.7	EG1 = Balance group EG2 = Walking group	“Light (11)” and “Somewhat hard (13)” according to the perceived exhaustion scale	EG1 = EG2 = 52/3-5/30-50	Significant improvements: 6MWT ↑, one-leg stance with eyes closed ↔.
Paillard et al. (26)	Pre-posttest	EG = 11 (M) CG = 10 (M)	EG = 65.5 ± 2 CG = 66.8 ± 2	EG = Walking program CG = No exercise	No present	EG = 12/5/36	Significant improvements: lateral dynamic balance ↑, fat mass ↓, VO_2_ max ↑.
Song et al. (44)	Pre-test, post-test 1, post-test 2, post-test 3	EG = 35 (F) CG1 = 35 (F) CG2 = 35 (F)	EG = 61.83 ± 4.37 CG1 = 62.85 ± 5.29 CG2 = 62.14 ± 5.52	EG = Tai Chi Chuan CG1 = Dance Group CG2 = Moderate walking	The exercise intensity is con- trolled to be medium.	EG = 52/6/40 CG1 = CG2 = 52/6/40	Significant improvements: Post-test 1, eyes closed and stepping in place ↑, Hip extension strength ↑, knee extension strength ↑.
Swoap et al. (24)	Pre-posttest	EG1 = 10 (M) and 14 (F) EG2 = 14 (F) and 12 (M) CG = 9 (M) and 9 (F)	EG = 65.2 ± 4.2	EG1 = High intensity exercise EG2 = Moderate intensity exercise group CG = No exercise control group.	Moderate intensity at 40–50% to 65–70%of maximum heart rate reserve High intensity at 40–50% to 80–85%maximum heart rate reserve	EG1 = 26/3/45 EG2 = 26/3/40	Significant changes: VO_2max_ ↑, body weight ↓, and body composition ↓.
Wanderley et al. (45)	Pre-posttest	EG = 22 (F)	EG = 71.4 ± 5.9	EG = Moderate-intensity Walking	50–70% of heart rate reverse	EG = 16/3/50	Significant changes: systolic blood pressure ↓, and muscular endurance ↑, in the lower limbs' strength ↑, and upper limbs' strength ↑, body composition (DXA ↔, flexibility ↔, dynamic balance ↔, and aerobic endurance ↔.
Yoo et al. (46)	Pre-post-test	EG = 11 (F) CG = 10 (F)	EG = 70.9 ± 2.7 CG = 71.1 ± 2.7	EG = Exercise group CG = No exercise group	60% of heart rate reverse	EG = 12/3/60 CG = 12/3/60	Significant changes: upper body strength (handgrip strength) ↑, leg strength ↑, aerobic endurance ↑, and body composition (DXA) ↓, balance ↔.

*EG, Experimental group; EG, Experimental group one; EG, Experimental group two; CG, Control Group; CG, Control Group one; CG, Control Group two; HRR, Heart rest rate; M, Male; F, Female; 6MWT, 6 min walk test; 6MWD, 6-min walking distance; KF, Muscle thickness for knee flexors; DF, Dorsi flexors; PF, Plantar flexors; DXA, Dual-energy X-ray Absorptiometry; ↑, significant improvement; ↓, significant decrease; ↔, no significant difference; SD, Standard deviation; Wk, week; f, frequency; min, minute*.

### Effect of Brisk Walking on Health-Related Physical Fitness, Balance, and Life Satisfaction Among the Elderly

The research results of 13 papers were considered and showed the impact brisk walking on health-related physical fitness, balance, and life satisfaction among the elderly. Table 3 summarizes the major studies included in this study.

### Effect of Brisk Walking on Cardiorespiratory Fitness

Ten publications discussed cardiorespiratory fitness (21–24, 26, 27, 46, 48). Among them, walking endurance, VO_2_ max, peak aerobic capacity, 6-min walk, and 6-min walking distance were used as measurement techniques. Among these, 3–7 times per week, 12–52 weeks of brisk walking (40–85% of heart rate reverse, 40–80% maximum heart rate, ≥3 metabolic equivalents (METs) daily (22), ~50% VO_2_ peak (27), “Light (11),” and “Somewhat hard (13)” (23) has a considerable effect on cardiorespiratory fitness. Six of them referred to females (18, 21, 22, 45, 46, 48), one referred to males (26), and three referred to both (23, 24, 27). Eight articles have concluded that there was a significant difference in the brisk walking group between the pre-test and post-test (21–24, 26, 27, 46, 48). Nevertheless, two articles showed no significant difference between the pre-test and post-test (18, 45).

### Effect of Brisk Walking on Body Composition

Four of the 13 articles discussed the subject of body composition (24, 26, 45, 46). Two trials tested body composition by dual-energy X-ray absorptiometry (DXA) (45, 46). Fat mass (24) and body weight (26) were also considered measurements. One article was devoted to males (26), two to females (45, 46), and another a mixture of both (24). Three articles concluded that brisk walking (The training time ranged from 12 to 26 weeks, 3–5 times per week, 36–50 min, the intensity at 40–85% of heart rate reverse) has a significant influence on body composition between the pre-test and post-test (24, 26, 46). Conversely, one paper showed that there was no significant improvement (45).

### Effect of Brisk Walking on Flexibility

One of the 13 publications included a section on flexibility (45). There was no significant difference on chair sit-and-reach between baseline and 4 months among elderly women. Scilicet, no effect of brisk walking (50 min, 3 times per week, 16 weeks, at 50–70% of heart rate reverse) on flexibility.

### Effect of Brisk Walking on Muscular Endurance

One of the 13 articles (45) established the concept of muscular endurance. Lower body muscular endurance (30 s chair stand) and upper body muscular endurance (30 s arm curl) were included in the test items (45). After 16 weeks of brisk walking (50 min, 3 times per week, at 50–70% of heart rate reverse), elderly women's lower limb muscular endurance improved, but there was no significant improvement in upper limb muscle endurance (45).

### Effect of Brisk Walking on Muscular Strength

Five of the 13 articles discussed muscular strength (18, 19, 27, 44, 46). Measurements included right knee extension and left knee extension (18, 19), left handgrip, right handgrip, upper body strength, leg strength (46), muscle thickness, knee extensors, knee flexors, dorsi (19), hip extension strength, and knee extension strength (44). Five articles demonstrated that there was significant improvement in lower body strength, while one article showed that there was significant improvement in upper body strength (46). The total intervention time ranged from 15–40 to 60 min and 3 to 6 times, 12 to 52 weeks at different intensity [50–70% of heart rate (18), ~50% VO_2_ peak (27), 60% heart rate reverse (46)]. Three studies focused on females (18, 44, 46) and two considered a combination of both males and females (19, 27). No studies considered only males.

### Effect of Brisk Walking on Balance

Six of the 13 papers discussed balance (18, 23, 26, 44–46). Balance is determined using a one legged balance with eyes closed and open (18, 23). Another study established that the best method for determining balance is to use a seesaw platform (26). There was no significant difference for balance in some of the studies (45, 46). There was significant difference in balance according to three studies (18, 26, 44). Nevertheless, there was no significant improvement in balance in three studies (23, 45, 46). The total time of intervention ranged from 30 to 60 min, 3 to 6 times per week, 12 to 52 weeks at intensity [50–70% of heart rate (18), 50–70% of heart rate reverse (45), Light (13), and Somewhat hard (15, 23)]. One study considered males (26), two considered females (18, 44), and three considered both males and females (23, 45, 46).

### Effect of Brisk Walking on Life Satisfaction

Only one article discussed the effect of brisk walking (30–40 min, 3 times per week, 24 weeks) on life satisfaction. Life satisfaction was tested by satisfaction with a life scale. There was a significant difference in life satisfaction between the pre-test and 6 months on males and females (25).

## Discussion

Previous studies have shown that health related physical fitness is a strong independent predictor of mortality (49–51). Consequently, each of its components is very essential for the elderly to consider. This study aimed to explore whether brisk walking improves health related physical fitness, balance, and life satisfaction among the elderly. Overall, Table 4 summarized the distribution of studies by component and the degree of scientific evidence according to risk of bias. The assessment of bias within brisk walking studies revealed that 11 were classified as low risk and two as high risk. Thus, based on the recognized criteria, there is compelling evidence that brisk walking improves cardiorespiratory fitness, body composition and muscular strength. There is limited evidence that brisk walking improves flexibility, muscular endurance, and life satisfaction, while one study reported conflicting results on balance.

**Table 4:** The study of brisk walking and health-related physical fitness components was classified according to the risk of bias within the studies and the strength of the scientific evidence.

**Health-related physical fitness component and balance**	**Studies that demonstrated association**	**Studies by risk of bias**	**Low risk of bias studies that showed significant association**	**Level of evidence**
Muscular strength (*N* = 5)	YES: 5 (100%)	Low: 5 (100%) High: 0	Yes: 5 (positive association) No:	Strong evidence
Muscular endurance (*N* = 1)	YES: 1 (100%)	Low: 1 (100%) High: 0	Yes: 1 (positive association) No:0	Limited evidence
Cardiorespiratory fitness (*N* = 10)	YES: 10 (100%)	Low: 8 (83.3%) High: 2 (16.7%)	Yes: 8 (positive association) No:2	Strong evidence
Body composition (*N* = 4)	YES: 4 (100%)	Low: 3 (75%) High: 1 (25%)	Yes: 3 (positive association) No:1	Strong evidence
Flexibility (*N* = 1)	YES: 1 (100%)	Low: 1 (100%) High: 0	Yes: 1 (positive association) No: 0	Limited evidence
Balance (*N* = 6)	YES: 6 (100%)	Low: 6 (100%) High: 0	Yes: 3 (positive association) No: 3	Conflicting evidence
Life satisfaction (*N* = 1)	YES: 1 (100%)	Low: 1 (100%) High: 0	Yes: 1 (positive association) No: 0	Limited evidence

Aging can cause changes in the heart and blood vessels that may increase a person's risk of developing cardiovascular disease, especially in the elderly (52). Cardiorespiratory fitness (CRF) reflects the functional capabilities of the heart, blood vessels, lungs, and skeletal muscles to perform work (53). A higher CRF is associated with improved survival and decreased incidence of CVD and other comorbidities, including hypertension, diabetes, heart failure, and atrial fibrillation (54). A previous study has shown that brisk walking can assist to improve cardiopulmonary fitness by increasing blood circulation, oxygen intake, and heart rate (55). Identically, this literature review revealed that moderate-intensity brisk walking had a positive and significant effect on cardiorespiratory fitness for elderly women and a mixture of males and females, but limited evidence presented on males (21, 22, 24, 26, 27, 46). High-intensity brisk walking for at least 60 min a week has a greater impact on cardiorespiratory fitness than those who walk at a leisurely-pace. However, with respect to moderate intensity activity, findings indicated that even when accumulated at high levels (i.e., ≥150 min/wk) did not result in significant improvements in cardiorespiratory fitness (56). Both brisk walking and resistance training (3 times a week at an intensity of 60–70% of their respective age-predicted maximum heart rate for 8 weeks) Can promote the cardiorespiratory fitness of the elderly has a more significant impact than brisk walking and resistance training (57). A number of studies have shown that brisk walking has a positive effect on cardiorespiratory fitness, among which high-intensity brisk walking has a more significant effect on cardiorespiratory fitness. Meantime, a study have shown that brisk walking combined with other exercises can promote cardiorespiratory fitness health greater than a single brisk walking, but this research still needs to be explored.

Aging causes changes in body composition, especially gradually increasing the obesity rate among the elderly (58). Studies have shown that the body composition anomalies are closely-related to lipid metabolic disorder, such as obesity, diabetes, and other diseases (59). Some research found that brisk walking has a beneficial and significant influence on body composition (24, 26, 46), whereas others found no effect on females (45). There is insufficient evidence regarding the effect of brisk walking on the body composition of elderly men (26), and mix of men and women (24), while there is a paradox on the effect of brisk walking on the body composition of women (45, 46). The heart rate should be between 75 and 80% of the maximum level. Brisk walking and similar activities should be no <30 min to elevate carbohydrates and fat utilization, and in turn alter the body's composition (60). Therefore, one study showed that walking at an intensity corresponding to 50–70% heart rate cannot influence body composition (45). As a result, the influence of brisk walking on body composition remains unknown and debated in the literature. Complementarily, the FITT of the brisk walking still need to be continuously proven the effects on body composition of the elderly.

Aging causes muscle mass and muscle strength to decrease (61). Improving muscular strength and endurance slows bone density and muscle loss and prevents osteoporosis and frailty in the elderly (61). Brisk walking stimulates leg muscle action, and when combined with the hip twist, it has a certain influence and promotion effect on the promotion of the lower limbs, waist, and abdomen strength (62). Therefore, brisk walking (above medium strength 50% of maximum heart rate) has a considerable effect on strength, specifically upper body strength (46), and lower limb strength (18, 19, 27, 44, 46), as well as on lower limb muscular endurance (45). Most studies indicated that brisk walking improves lower limb strength, but there remains insufficient evidence for upper body strength. Moreover, no study investigated the effect of brisk walking on elderly males. The effect in strength and muscular endurance of the upper limbs, and endurance of the lower limbs, remains unreported.

Falls account for 49.3% of all accidental injuries (7). It is a complicated issue that the elderly must address. Balance exercises are critical in avoiding elderly people from falling (63). A previous study identified that brisk walking benefits balance (64). Similarly, this study confirms that brisk walking improves balance (18, 26, 44). Nonetheless, there was no influence on balance by some studies (45, 46). Intervention on studies do not continuously lead to the outcome (23). Additionally, a small sample size also influences the results (45). Hence, there was inconsistency on the influence of balance in this study. Furthermore, further research is required to confirm the most effect of brisk walking on balance at which level of FITT.

Aging causes the loss of a small amount of flexibility because of the natural aging processes. This can occur for several reasons, including loss of water in the tissues and spine, increased stiffness in joints and a loss of elasticity throughout the muscle tendons and surrounding tissue (65). There is no stretching prior to and during brisk walking exercise, hence brisk walking is unlikely to result in changes in flexibility (66). As a result, it was concluded that 12 weeks of brisk walking did not improve flexibility in the elderly (45). Exploring the impact of brisk walking on the flexibility of the elderly is critical in future work.

Among older adults, life satisfaction correlates with health, mortality and successful aging especially with advancing years (67). Previous work reported that physical activity was significantly related to life satisfaction and happiness in older adults (68). Similarly, life satisfaction was improved in brisk walking intervention after 6 months (25). However, the evidence for a linkage of physical activity levels to life satisfaction or is not always positive (Evidence Category C) (69). Life satisfaction is a measure of well-being assessed in terms of mood, satisfaction with relationships, achieved goals, self-concepts, and self-perceived ability to cope with one's daily life (70). Additionally, satisfaction with the body, social or family relationships, and financial circumstances may all contribute to global life satisfaction. Satisfaction with physical function and appearance may also be important when judging levels of life satisfaction due to cultural prominence of certain body types (71). In aging, the health status of the elderly has the most important impact on life satisfaction (72). Consequently, life satisfaction is a relatively complicated item to evaluate. Considering whether the community-level physical activity intervention has an impact on the life satisfaction of the elderly is obviously insufficient and only one study showed this result. Thence, considering sundry factors to check the impact of brisk walking on life satisfaction is urgent in this field.

The results indicated that subjects in the high intensity (80–85% of maximum heart rate) exercise group have a significant improvement on aerobic capacity than moderate intensity (65–70% of maximum heart rate) of the maximal heart rate reserve exercise group (24). High-intensity (low-intensity walking intervals at ~40% of the pre-training VO_2peak_, followed by a 3-min interval of high-intensity walking >70% but <85% VO_2_ peak for walking) 40-min intermittent brisk walking which undergo 3 times per week and 26 weeks activities performed daily had a greater effect on the elderly's aerobic capacity than moderate-intensity (Walking more than 8,000 steps per day at ~50% VO_2peak_) 50-min intermittent brisk walking exercises (27). WHO recommend that 150–300 min of moderate-intensity aerobic physical activity; or at least 75–150 min of vigorous-intensity aerobic physical activity or an equivalent combination of moderate- and vigorous-intensity activity throughout the week, as these provide additional health benefits (8). Moreover, high-intensity cardiorespiratory exercise has a more significant impact on metabolic syndrome (MS) than low- and medium-intensity in sedentary, overweight, moderately hypertensive, post-menopausal women (73). However, moderate activity increased the probability of successful aging of the elderly by 0.76–0.78% (*P* < 0.001), while participation in vigorous and mild physical activity had no significant effect on successful aging (*P* > 0.05) (74). Therefore, according to most studies, high-intensity exercise has a better effect than low-medium-intensity exercise, but some health factors in the elderly require moderate exercise intensity, and research in this area is still to be studied.

### Study Limitations

Overall, this review demonstrated the efficacy and favorable benefits of brisk walking on health-related physical fitness, balance, and life satisfaction in the elderly. This systematic review, however, has a few drawbacks. First, the sample size of the included studies is often small. Seven studies used a sample size of fewer than 30 participants in each category (18, 22, 24–26, 45, 46). Second, most of the studies focused on female health-related physical fitness balance and life satisfaction (18, 21, 22, 44–46, 48), only two studies focus on males (23, 26). Finally, the research had some significant flaws. Simultaneously, the measurement methods of exercise intensity are inconsistent, so that difficult to judge which intensity has the greatest impact on the elderly. There is no research on all indications of health-related physical fitness in the literature, although most studies focus on a single health-related component. There is only one paper on flexibility (45), muscular endurance (45), and life satisfaction (25), and it has a low level of credibility and persuasiveness. Although there are numerous publications devoted to muscular strength research, there is only one devoted to the same index of upper body strength (46).

## Conclusion

This systematic review demonstrates that brisk walking improved cardiorespiratory fitness, muscular strength, and body composition among the elderly. There are less studies on the effects of flexibility, muscular endurance, life satisfaction, and additional research is necessary to demonstrate the effects of these three components. Additionally, conflicting evidence on balance should be confirmed by further research. Moreover, according to most studies, high-intensity exercise has a better effect than low-medium-intensity exercise but some study showed the most impact should be an apposite intensity. The measurement methods of exercise intensity are different, and it is impossible to accurately summarize which training principle of FITT has the safest and most comfortable effect on the elderly. Sum up, the research evidence of brisk walking is still insufficient and single brisk walking cannot satisfy the requirements of the elderly in terms of health-related physical fitness, balance, and life satisfaction. According to the ACSM, a combination of several exercise modalities can effectively boost the health of the elderly. Future research can discover multiple types of exercise methods to promote the health-related physical fitness, balance, and life satisfaction of the elderly. Additionally, the induction of brisk walking training principles of FITT is very vital for the health effects among the elderly. Hence, this is also a potential research that deserves to be unearth.

## Data Availability Statement

The original contributions presented in the study are included in the article/supplementary material, further inquiries can be directed to the corresponding authors.

## Author Contributions

The literature search, selection of studies, and study quality assessment was performed by XB and WX. Following an initial screen of titles and abstracts, full scrutiny of potentially eligible studies was independently screened by XB and WX using the specific inclusion criteria. KS and RO arbitrated any disagreements in study inclusion. OT and HC arbirated any disagreements in assessment study quality. All authors contributed to manuscript revision, read, and approved the submitted version.

## Conflict of Interest

The authors declare that the research was conducted in the absence of any commercial or financial relationships that could be construed as a potential conflict of interest.

## Publisher's Note

All claims expressed in this article are solely those of the authors and do not necessarily represent those of their affiliated organizations, or those of the publisher, the editors and the reviewers. Any product that may be evaluated in this article, or claim that may be made by its manufacturer, is not guaranteed or endorsed by the publisher.
